# Inflammatory microenvironment in gastric premalignant lesions: implication and application

**DOI:** 10.3389/fimmu.2023.1297101

**Published:** 2023-11-15

**Authors:** Shengxiong Zhang, Yang Shen, Hao Liu, Di Zhu, Jiansong Fang, Huafeng Pan, Wei Liu

**Affiliations:** ^1^ Rehabilitation Department, Guangdong Work Injury Rehabilitation Hospital, Guangzhou, China; ^2^ Department of Spleen and Stomach, GuangZhou Tianhe District Hospital of Chinese Medicine, Guangzhou, China; ^3^ The Second Affiliated Hospital, Guangzhou University of Chinese Medicine, Guangzhou, China; ^4^ The First Affiliated Hospital, Guangzhou University of Chinese Medicine, Guangzhou, China; ^5^ Science and Innovation Center, Guangzhou University of Chinese Medicine, Guangzhou, China

**Keywords:** gastric precancerous lesions, inflammatory microenvironment, gastric cancer, antioxidants, TCM

## Abstract

Gastric precancerous lesions (GPL) are a major health concern worldwide due to their potential to progress to gastric cancer (GC). Understanding the mechanism underlying the transformation from GPL to GC can provide a fresh insight for the early detection of GC. Although chronic inflammation is prevalent in the GPL, how the inflammatory microenvironment monitored the progression of GPL-to-GC are still elusive. Inflammation has been recognized as a key player in the progression of GPL. This review aims to provide an overview of the inflammatory microenvironment in GPL and its implications for disease progression and potential therapeutic applications. We discuss the involvement of inflammation in the progression of GPL, highlighting *Helicobacter pylori* (*H. pylori*) as a mediator for inflammatory microenvironment and a key driver to GC progression. We explore the role of immune cells in mediating the progression of GPL, and focus on the regulation of inflammatory molecules in this disease. Furthermore, we discuss the potential of targeting inflammatory pathways for GPL. There are currently no specific drugs for GPL treatment, but traditional Chinese Medicine (TCM) and natural antioxidants, known as antioxidant and anti-inflammatory properties, exhibit promising effects in suppressing or reversing the progression of GPL. Finally, the challenges and future perspectives in the field are proposed. Overall, this review highlights the central role of the inflammatory microenvironment in the progression of GPL, paving the way for innovative therapeutic approaches in the future.

## Key points

• Chronic inflammation stimulates cells to secrete inflammatory factors and changes in immune cell function, which further promotes inflammatory changes in the gastric mucosa and even leads to cancer.
• Altered inflammatory immune microenvironment due to *H. pylori* infection enhances cytogenic Correa cascade progression based on epidemiological investigation and basic research.
• Inflammatory molecules regulation in gastric precancerous lesions progression.
• Traditional Chinese Medicine (TCM) and natural antioxidants, known as antioxidant and anti-inflammatory properties, exhibit promising effects in suppressing or reversing the progression of GPL.

## 1 Introduction

Gastric cancer(GC) ranks as the fifth most common tumor globally and stands as the third leading cause of cancer-related mortality across the world (1, 2) Its incidence is most pronounced in East Asia (3). Notably, GC is twice as likely to afflict men compared to women (1). The prognosis for advanced GC, with a 5-year survival rate of under 20%, is grim, while early gastric cancer (EGC) enjoys a favorable outlook, boasting a 5-year survival rate ranging from 90% to 95% (4).

Several prominent risk factors for GC encompass *H. pylori* infection, age, and dietary patterns. The progression of gastric lesions, from superficial gastritis (SG) to chronic atrophic gastritis (CAG), spasmolytic polypeptide-expressing metaplasia (SPEM), intestinal metaplasia (IM), and low-grade intraepithelial gastritis neoplasia (LGIN), can eventually lead to high-grade intraepithelial neoplasia (HGIN) and aggressive GC (5). These precancerous states (CAG, SPEM, and IM) and precancerous lesions (LGIN and HGIN) are associated with an elevated risk of GC. Chronic infection of the gastric mucosa lays the foundation for the progression of CAG and IM to gastric mucosal cancer. Approximately 5% of dysplasia (Dys) patients develop GC within two decades (6). Evidence suggests that IM arises from SPEM in humans, indicating that SPEM is the key initial pretumor metaplasia in gastric adenocarcinoma. Biopsies obtained before cancer diagnosis have shown that SPEM was detected in more than 4 out of 5 tumor patients, compared to only 1 out of 3 gastritis patients (7).

The clinical presentation of GPL is marked by nonspecific manifestations, including upper abdominal discomfort, acid reflux, and nausea, among others. It should be noted that there is no clear correlation between the severity of the pathology and the symptoms. The development of precancerous lesions is closely related to inflammatory processes and immune responses. Assessing the immune molecule expression in GPL is pivotal in evaluating the inflammatory status. Nevertheless, inflammatory response and immune response in GPL still poorly elucidated. On the other hand, treatment options for GPL encompass surgical intervention, *H. pylori* eradication, cyclooxygenase-2(COX-2) inhibitor and other symptomatic treatment, without specific therapy. However, traditional Chinese medicine and its active ingredients are effective in the treatment of GPL and more and more related studies, but there is a lack of systematic summary. This review aims to provide a concise overview of our current comprehension of diverse inflammatory immune response, and the treatment of traditional Chinese medicine and its active ingredients, unveiling the most recent research findings regarding their potential mechanisms of GPL.

## 2 Inflammation participates in the gastric precancerous lesion progression

Substantial epidemiological evidence has demonstrated that chronic inflammation of the gastric epithelium is important in GC development. This connection between inflammation and GC in humans has been meticulously documented through lifelong studies by Correa, which documents a clear association between inflammation and GC. Inflammatory microenvironments are common pathological characteristics and drive the development of multiple GPL (8). The cells and mediators responsible for inflammation constitute a substantial portion of the epithelial inflammatory microenvironment. In GPL-to-GC, inflammatory conditions often precede the onset of malignancy. Moreover, oncogenic changes create a tumor-promoting inflammatory milieu (9).

In 1994, *H. pylori* was unequivocally designated as a Class I carcinogen with a proven link to GC (10). The infection statistics reveal that 1%-3% of individuals harboring *H. pylori* will ultimately develop GC (11). This persistent infection takes root by inciting chronic and active inflammation within the gastric mucosa, setting the stage for a cascade of pathological events. The stepwise progression from *H. pylori*-induced GPL-to-GC has been meticulously defined in various animal models, including mice and Mongolian gerbils (12). The stomach’s enduring inflammation often triggers metaplastic alterations in the mucosa, characterized by the atrophy of mature oxyntic cells and the emergence of novel metaplastic lineages. Consequently, the degenerating mucosa can adopt a more proliferative phenotype, substantially elevating the risk of GPL transitioning to GC (13).

*H. pylori*, which harbors the cag pathogenicity island, triggers immune cell infiltration. Paradoxically, this robust immune and inflammatory response fails to eradicate the infection, leaving the host gastric mucosa ensnared in the enduring throes of inflammation. *H. pylori* infection stimulates inflammation and altered immune cell function promoting malignant transformation of GPL, according to growing evidence. Inflammatory response on gastric from *H. pylori* infection shows “*sui generis*” characteristics that are scarcely observed in other organs (14). Furthermore, research has unveiled that an immune response induced by Helicobacter pylori can promote genetic changes. The alterations encompass changes in transcription factors (CDX2, RUNX3, TLR1), interleukins (IL1β, IL8), and the generation of oxidative stress-induced DNA damage. These genetic modifications activate genes that drive tumor development while concurrently suppressing tumor suppressor genes (15), essentially placing GPL patients in a chronic inflammatory state (16). It’s important to note that while cell proliferation alone does not inevitably lead to cancer, within the context of an inflammatory microenvironment teeming with inflammatory cells and growth factors, heightened cell proliferation undeniably amplifies the risk of tumorigenesis (17). Oxidative damage and DNA damage accumulate gradually along GPL-GC (18). With inflammation, parietal cells undergo apoptosis, paving the way for the emergence of CAG and metaplastic cells (19). The gastric environment accumulates a mass of immune cells, which in turn, produce a multitude of inflammatory cytokines. Immune molecules triggered by antigen stimulation, including antibodies, complement, and lymphokines, assume pivotal roles. Dysregulation of immune cell activity or imbalances in immune-related factors can yield profound consequences in this intricate interplay of factors.

## 3 Immune cells mediated gastric precancerous lesions progression

**Figure 1:**
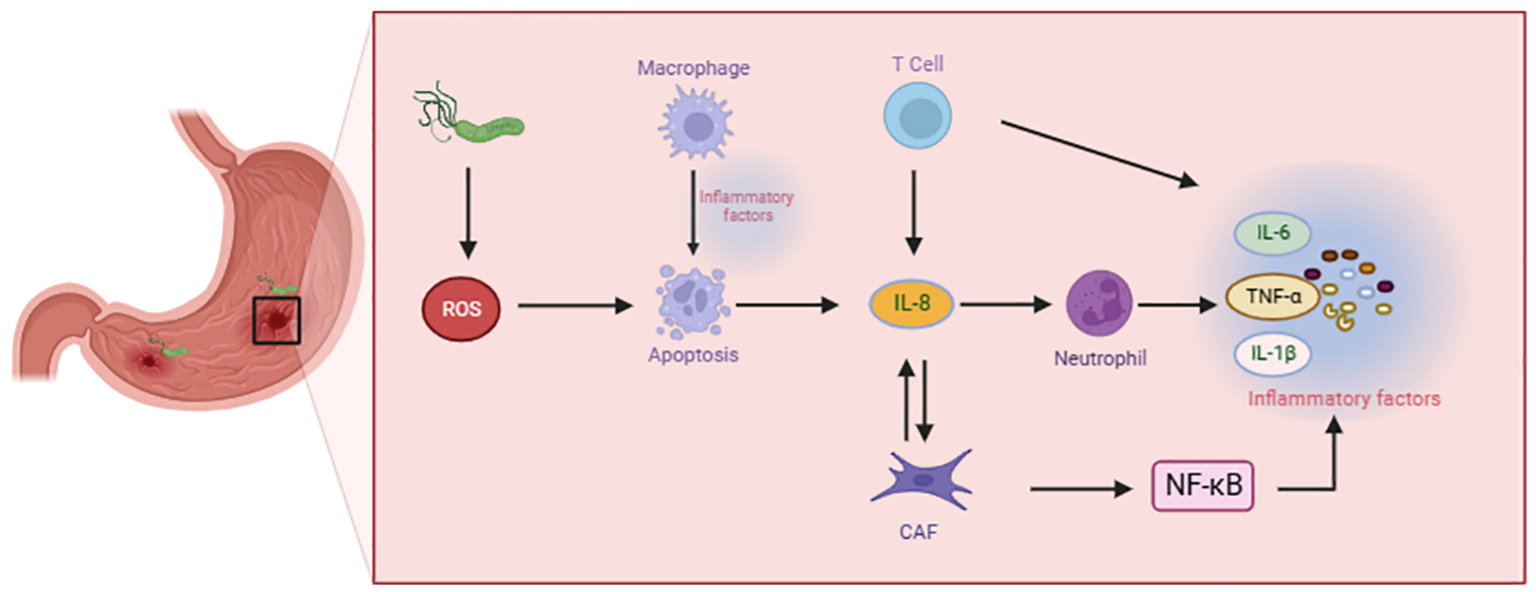
Immune cells contribute to the advancement of gastric precancerous lesions.

Malignant tumor cells thrive within a multifaceted cellular microenvironment, comprising a dynamic interplay of endothelial cells, fibroblasts, and an array of immune cells. This diverse cast of immune cell types encompasses innate immune cells, adaptive immune cells, and immunosuppressive cells. The insidious influence of H. pylori infection amplifies the production of cytokines, thereby instigating a cascade of events that recruit and activate immune cells within this intricate milieu (20). Neutrophils and sometimes eosinophils represent the initial responders in the acute inflammatory reaction. Following neutrophils infiltrate, immune cells are summoned to the site of injury or infection. Inflammation is critical to orchestrating the migration and function of macrophages and T cells. As the inflammatory response concludes, both macrophages and T cells must adopt a pro-resolving phenotype to gradually terminate the inflammatory process. But under specific conditions, immune responses and inflammation persist, leading to the progression of chronic inflammatory diseases (**Figure 1**).

### 3.1 Neutrophil immune regulation in gastric precancerous lesions progression

Neutrophil infiltration is a typical occurrence in cases of acute gastritis induced by *H. pylori (* 21). In the realm of tissue pathology, the extent of neutral granulocyte infiltration serves as a key indicator of gastritis inflammation severity (22). Typically, neutral granulocytes predominantly infiltrate the proliferation zone in normal conditions. However, in specific situations, they can extend into the surface region, potentially leading to the development of depressed abscesses (23). Throughout this process, neutral granulocytes may undergo apoptosis and be phagocytosed by foveolar cells (24).

The neutrophil recruitment is often attributed to the signaling of endogenous and bacterial chemoattractants (25). Furthermore, immune and epithelial cells participate in the immune response which triggered by *H. pylori* infection. Some types of inflammatory factors (such as IL-8, IL-1β and tumor necrosis factor-α(TNF-α)) are the major participants of the reaction. These factors can stimulate IL-8, causing neutrophil infiltration and the exacerbation of inflammation (26). Research has established that neutrophils activated by the water-soluble surface proteins of *H. pylori (* 27). Furthermore, nicotinamide adenine dinucleotide phosphate (NADPH) oxidase subunits in *H. pylori*’s fatty polysaccharides within neutrophils can lead to an overwhelming generation of reactive oxygen species (ROS), thereby promoting inflammatory damage (28). In this process, the protein domain 3 of ARRDC3 facilitates the accumulation and migration of neutrophils in the gastric mucosa (29). Scientific investigations have found that neutrophil extracellular traps (NETs) can enhance more aggressive mesenchymal phenotypes, thereby contributing to GC progression both *in vitro* and *in vivo*. Targeting NETs holds promise as a potential therapeutic approach (30). Moreover, the neutrophil-to-lymphocyte ratio has emerged as a potential prognostic indicator for cancer, owing to its ease of acquisition in clinical settings (31). Preoperative NLR serves as an independent prognostic factor for GC patients, providing stratified prognostic value, especially in cases classified as AJCC stage III (32).

### 3.2 T Lymphocyte immune regulation in gastric precancerous lesions progression

The immune response of the human body to *H. pylori* is a multifaceted and constantly evolving process. During childhood, notable features include a significant increase in FoxP3+ Treg cells within the gastric mucosa, along with substantial elevations in the levels of Treg, Transforming growth factor beta 1 (TGF-β1), and IL-10. This pattern stands in stark contrast to the immune response observed in infected adults (33). In the case of adults, diverse scenarios unfold. The gastric mucosa exhibits a TH1 reaction and TH17 reaction, characterized by a reduction in TGF-β1 concentration and an upsurge in IFN-γ, IL-12P70, IL-17A, IL-23, and other cytokines. Of particular interest is the synergy between TGF-β1 and IL-6, which collaboratively promote the expression of IL-23, thereby enhancing the TH17 response. This unique immune reaction is intricately linked to the damage observed in gastric mucosal cells. Consequently, adults are more predisposed to the progression of GPL (34–36).

In patients with GC, specific subgroups of immune cells within tumor tissues are associated with prognosis. Tumors characterized by high expression of CD8 (+) cytotoxic T lymphocytes often correlate with a favorable prognosis. Conversely, the risen of Foxp3 (+)/CD8 (+) and Foxp3 (+)/CD4 (+) ratios may serve as indicators for a poorer prognosis (37).

### 3.3 Macrophages immune regulation in gastric precancerous lesions progression

Monocytes and macrophages initiate from bone marrow progenitor cells before entering the bloodstream. In response to inflammation, circulating monocytes respond to local growth factors by migrating into tissues, where they can differentiate into macrophages. Guided by chemotactic factors, tissue-resident macrophages then migrate to the site of tissue damage (38). In 1908, Elie Metchnikoff and Paul Ehrlich made pioneering observations concerning macrophages and their phagocytic activity. Macrophages play a pivotal role as the immune system’s first line of defense, contributing to the defense against infection by generating pro-inflammatory factors, including IL-1β (39). Activated macrophages serve as the primary source of growth factors and cytokines, exerting profound effects on local mucosal tissues and thus shaping the chronic inflammatory microenvironment (40). In cases of mild gastritis, macrophages infiltrate the stomach after parietal cell loss, promoting metaplasia progression (41). However, macrophages also exhibit specialization in response to local environmental cues, resulting in distinct gene expression profiles and functions across various organ systems (42). M2 macrophages, driven by Th2 cytokines, are characterized as anti-inflammatory tumor-associated inflammatory cells, which can detrimentally influence gastric tumors in GPL mice (43). Inflammatory process in gastric is associated with an increase of secretory activity of macrophages. This heightened M1 macrophage activation can exacerbate gastric inflammation and result in a reduced bacterial load (44). Studies have demonstrated that in mouse model tissues, M2 macrophages can promote cellular SPEM by inflammatory stimulation (41). Moreover, macrophages possess the capability to release cytokines and chemokines into the bloodstream, including IL-1β, TNF-α, IL-6, IFN-γ, and PGE2 (45), contributing to systemic chronic inflammation. It is widely believed that inflammation underlies gastric dysfunction. In GC, macrophage polarization transitions from the anti-inflammatory M1 state to the pro-inflammatory M2 state. Pathogens such as *H. pylori* can impede the M1 macrophage response, induce macrophage polarization into the M2 state, and increase ROS-induced macrophage apoptosis, thereby advancing the progression of GPL (44).

However, in a persistent inflammatory state, immune functions can become detrimental, leading to the production of mutagenic agents like peroxynitrite, which can react with DNA, promoting uncontrolled division of epithelial and stromal cells. Macrophage infiltration can release TNF-α, exacerbating DNA damage, potentially linked to IL-33 (43). Notably, *H. pylori* induces apoptosis in macrophages and is a strategy to escape the immune response. Phagocytosis of *H. pylori* triggers apoptosis in macrophages, releasing bacteria to infect the next cell (46, 47).

### 3.4 Fibroblasts immune regulation in gastric precancerous lesions progression

Stromal fibroblasts are pivotal contributors to the intricate web of chronic cancer-related inflammation and the initiation and progression of malignant diseases (48). Fibroblasts can produce IL-6, thereby inducing TNF, IL-17, IL-1β, LPS and IFNs (49). In addition, *H. pylori* increases caspases and sST2, causing deleterious effects on gastric barrier cells. Gastric epithelial cells and fibroblasts can upregulate type I collagen and repair early cell damage caused by *H. pylori* (50). Various immune cells circulate in the blood in response to specific environmental signals. When the gastric mucosa is damaged, these immune cells will be recruited to the damaged tissue, promote the formation of new blood vessels, and create an immunosuppressive environment. Studies have found that cancer-associated fibroblasts (CAFs) participate in ECM remodeling and promote angiogenesis, leading to the progression of GPL (51). CAFs are also capable of secreting miR-522 to suppress ferroptosis and bolster angiogenesis (52). Furthermore, CAFs-derived IL-8 amplifies the inflammatory response by activating signaling pathways such as nuclear factor kappa-B (NF-κB) (53). Additionally, *H. pylori* infection has been shown to induce the transformation of fibroblasts into myofibroblasts, elevating the early oncogenic marker HIF-1α (54). *H. pylori*-activated gastric fibroblasts are central to promoting the transition of normal gastric epithelial cells into a precancerous state, driving EMT through the regulation of TGFβ R1/R2-dependent signaling. In summary, *H. pylori* infection intensifies CAFs differentiation, subsequently promoting EMT through pathways involving NF-κB, STAT3, and TGF-β. Given the pivotal role of CAFs in the microenvironment of gastric, targeting CAFs emerges as a potential strategy for enhancing patient prognosis (53, 55).

The mechanism of GPL is correlated with neutrophils, T cells, macrophages and fibroblasts. As a key inflammatory factor, IL-8 interacts with different immune cells, triggering the release of more inflammatory factors and aggravating the cascade reaction of GPL.

## 4 Inflammatory molecules and microorganisms regulation in gastric precancerous lesions progression

In the progression of gastric precancerous lesions, the regulation of inflammatory molecules and microorganisms plays a crucial role. Several types of inflammatory molecules have been implicated in this process, including the gut microbiome, bile acid, and cytokines (**Table 1**). Understanding the intricate interplay between these components is crucial for comprehending the mechanisms underlying GPL and may pave the way for novel therapeutic interventions.

**Table 1:** Summary of inflammatory molecules and microorganisms implicated in gastric precancerous lesions.

Name	Type	Function description	Implicated in GPL	PMID
Gut microbiome	Microorganism	Digestion, development, fecundity, and lifespan	Impact immune responses and inflammatory cytokines release	30510004
Bile acid	Endogenous metabolites	Dissolve lipid, modulate hepatic and intestinal functions and improve insulin sensitivity	Stimulate macrophages, release exosomes	32033746
IL-8	Chemokine	Mediate the inflammatory response	lead to DNA and tissue damage	14760971
IL-1β	pro-inflammatory cytokine	Stimulate the synthesis of prostaglandins, activate neutrophils, T-cell and B-cell	Recruit and activate immune cells	17676045
IL-33	Cytokine	Initiate the release of T-helper type 2-associated cytokines	Upregulate cells apoptosis, increase caspase-3, decrease Bcl-xL	32151084
iNOS	messenger molecule	Produce nitric oxide and involve in inflammation	Modulate the inflammation	10348815
COX-2	enzyme	An enzyme that is phosphorylated by oxidation	Modulate the inflammation	10348815

### 4.1 Gut microbiome implicated in gastric precancerous lesions progression

The equilibrium of the intestinal microbiota is intricately intertwined with the host’s well-being. The presence of *H. pylori* can disrupt the balance of the intestinal flora, thereby fostering the advancement of GPL. This phenomenon is intricately linked to the persistent activation of the host’s immune system by the intestinal microbiota, which, in turn, results in localized chronic inflammation. On one hand, the intestinal flora is central to regulating anti-tumor immune responses, while, on the other hand, it can facilitate the generation of carcinogenic metabolites. An abnormal immune response can precipitate an imbalance in the intestinal flora, ultimately leading to an abnormal release of inflammatory factors (56). Therefore, elimination of *H. pylori* can correct intestinal flora disorder and have a healthy impact on the gastrointestinal tract (57). GPL progression is related to the abundance of *H. pylori* and other gastrointestinal flora. This suggests that intestinal dysbiosis has the potential to serve as a biomarker to differentiate between gastritis and GC (58, 59). This has been confirmed both in rat models and in human tissues, where the changes observed are very similar (60). One thing needs attention, although the infection abundance of *H. pylori* gradually increases in different stages of GPL, GC has less *H. pylori*, and the bacterial flora is dominated by oral and intestinal pathogenic microbial strains (61). Furthermore, IM patients are colonized with abundant oral bacterial genera, including Peptostreptococcus oralis, Neisseria elongatus, Johnsonella martensi, and Neisseria flavus (62). In addition, Acinetobacter may promote the development of intraepithelial neoplasia. Certain bacterial genera show a higher degree of centrality in the progression of GPL, such as gastric mucosal genera (including Gemini, Streptococcus, etc) (59). The gut microbiota and its metabolites may be central to the progression of GPL. Studies have shown that *H. pylori* can regulate gut microbiota. This regulate may include species changes in the microbiota, metabolites of the microbiota. And intestinal microbiota in turn can regulate the inflammatory immunity of gastric mucosa, resulting in the progression of GPL. This interaction between Helicobacter pylori and the gut microbiota can be referred to as the Helicobacter pylori-Gut microbiota Metabolism (HGM) axis (60, 63, 64).

### 4.2 Bile acid in gastric precancerous lesions progression

Bile acid reflux is a critical factor in the occurrence of gastrointestinal metaplasia (GIM), and this pathogenesis does not require the involvement of *H. pylori* (65, 66). The underlying mechanism involves deoxycholic acid stimulating macrophages to release exosomes encapsulating inflammatory factors (67). These exosomes, in turn, promote the overexpression of hsa-miR-30a-5p in gastric mucosal epithelial cells. Overexpression of this miRNA targets Forkhead Box D1 (FOXD1) and leads to the increased expression of CDX2, thereby promoting the development of intestinal metaplasia and GIM (68). The possible mechanisms of bile acid-induced gastritis have been documented, but the final substrates are all related to changes in miRNA and CDX2 substrates, which activate the expression of KLF4, cadherin 17, and HNF4α, leading to the progression of IM to GC (69–71).

### 4.3 Cytokines and inflammatory factors

**Figure 2:**
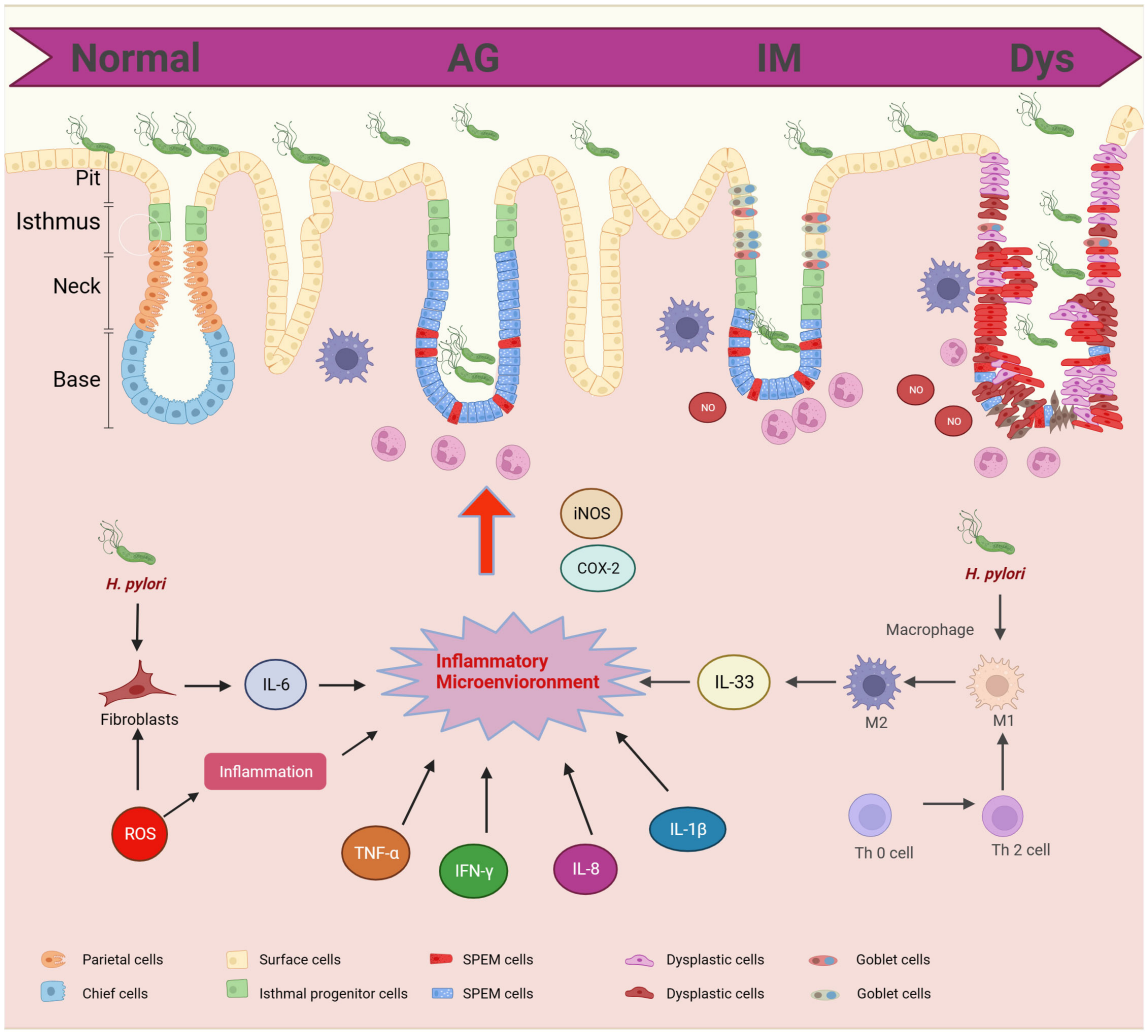
Cytokines and inflammatory factors in gastric precancerous lesions progression. GPL includes different pathological stages: AG, IM, and Dys. The pathological morphology is related to the inflammatory microenvironment. H.Pylori and T cells can promote the polarization of macrophages and induce the release of IL-33. Fibroblasts promote the release of IL-6 and ROS promotes the release of hormones. The inflammatory microenvironment promotes the infiltration of neutrophils into epithelial cells.

The inflammatory microenvironment is central to the progression of GPL to GC. Cytokines and inflammatory factors are central to immune response and are associated with a multitude of pathological changes associated with GPL (**Figure 2**).

#### 4.3.1 Interleukin-8 in gastric precancerous lesions progression

Interleukin-8 (IL-8), an important member of the CXC chemokine family, is a potent chemoattractant for neutrophils and lymphocytes and is critical to promoting gastric mucosal inflammation. IL-8 expression is significantly elevated in *H. pylori*-associated gastritis. IL-8 causes sustained overproduction of nitric oxide, which may induce DNA and tissue damage, thereby increasing the risk of neoplastic transformation (72). In histomorphology, IL-8 complements established predictors such as gastrin and pepsinogen A/C ratio (73). Highly expressed IL-8 can continuously infiltrate tissues and increase VEGF levels, leading to GPL (74).

#### 4.3.2 IL-1β in gastric precancerous lesions progression

Gastric IL-1β is closely linked to high grade mucosal inflammation (75), and is critical to the progression of CAG to GC (76, 77). The IL-1β exhibits polymorphic characteristics that are significantly linked to gastric acid secretion and GPL (78). IL-1β may collaborate with other inflammatory cytokines, such as promoting the upregulation of IL-17A, recruiting and activating immune cells within the gastric mucosa, collectively inciting inflammation (79, 80). Furthermore, IL-1β has been demonstrated to establish a positive feedback loop, inducing the expression of IL-8. It is noteworthy that while gastritis occurrence is associated with IL-1β and IL-18, exhibiting a declining trend from chronic gastritis to GC (81).

#### 4.3.3 Interleukin -33 in gastric precancerous lesions progression

Interleukin-33 (IL-33) is a recently characterized alarmin with high expression levels in the gastric mucosa, capable of potently activating Th2 immunity. after exposure to *H. pylori*, silencing IL-33 in GES-1 cells has been shown to lead to decrease cell metabolic activity, migration, adhesion and proliferation. Additionally, it resulted in an upregulation of cell apoptosis, marked by an increase in caspase-3 activity and a decrease in Bcl-xL expression. The findings suggest a proregenerative role of IL-33 (82). *H. pylori* infection can activate IL-33 pro-regenerative activity in apoptotic gastric tissue cells (83).

#### 4.3.4 Inducible nitric oxide synthase and cyclooxygenase-2 in gastric precancerous lesions progression

In patients with gastritis, especially those infected with *H. pylori*, nitric oxide produced by inducible nitric oxide synthase(iNOS) and COX-2 is induced to regulate epithelial cell growth and inflammatory changes (84). Studies have underscored the significance of iNOS as an inflammation-inducing enzyme and a key contributing factor to gastritis (85). Furthermore, iNOS can bind to *H. pylori* and induce apoptosis in gastric mucosal. Study shown that iNOS-KO mice exhibited persistent inflammation but not apoptosis after *H. pylori* infection (86).

There are many other cellular inflammatory molecules involved in the progression of GPL, and their mechanisms of action still need to be further explored. The involvement of inflammatory molecules may be closely related to the mechanism of action of immune cells.

## 5 Inflammatory pathways and TCM intervention in the prevention and treatment of GPL

**Figure 3:**
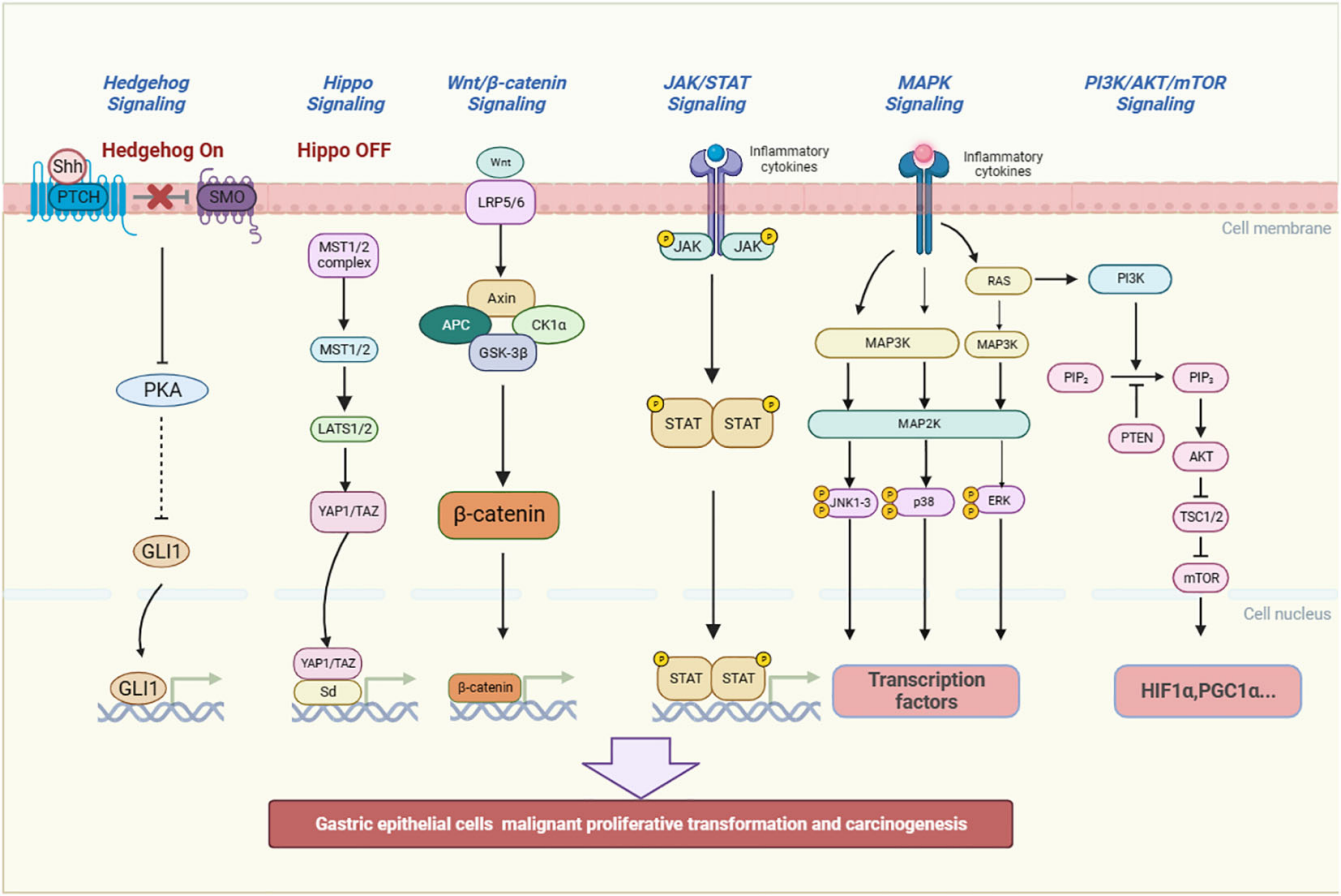
The inflammatory pathways related to the occurrence and development of GPL. They include MAPK, JAK/STAT3, PI3K/AKT/mTOR, the Hippo, Hedgehog and Wnt/β-catenin signaling pathway. The signaling pathways can promote gastric epithelial cells to undergo malignant proliferative transformation and carcinogenesis. (Shh, Sonic hedgehog, PTCH, Patched, SMO, Smoothened, PKA, Protein Kinase A, GLI1, glioma-associated oncogene homolog 1, MST, Mammalian Sterile 20-like kinase 1/2, LATS 1/2:large tumor suppressor kinase 1/2, YAP/TAZ, Yes-associated protein/transcriptional co-activator with PDZ-binding motif, Sd, Scalloped, Wnt, Wingless and INT-1, Axin1, Axis inhibition protein 1, APC, Adenomatous polyposis coli, CK1α:casein kinase 1α, GSK-3β:Glycogen synthase kinase 3β, JAK, Janus Kinase, STAT, Signal transducer and activator of transcription, RAS, Reliability, Availability and Serviceability, MAPK, Mitogen-activated protein kinase, JNK, c-Jun N-terminal kinase, p38:Peroxidase 38, ERK, Extracellular regulated protein kinases, PI3K:Phosphatidylinositol-3-kinase, PIP2:Phosphatidylinositol(4,5)bisphosphate, PTEN, Phosphatase and tensin homolog, AKT, Protein kinase B, TSC1/2:Tuberous sclerosis 1/2, mTOR, mammalian target of rapamycin, HIF1α:Hypoxia-inducible factor-1α, PGC1α, Peroxisome proliferator-activated receptor-γ coactivator 1α).

In the process of inflammatory stimulation of gastric mucosa leading to GPL, it is closely related to some inflammatory pathways, including classic pathways: MAPK, Wnt/β-catenin, JAK/STAT3 and PI3K/AKT/mTOR signaling pathway (87), and non-classic inflammatory pathways: the Hippo and Hedgehog signaling pathway (**Figure 3**). Traditional Chinese medicine (TCM) and TCM-derived natural products, known as antioxidant and anti-inflammatory properties, can interfere with the outcome of GPL by affecting the above pathways (**Table 2**).

**Table 2:** Summary of mechanism of traditional Chinese medicine targeting inflammatory pathways in the Prevention and Treatment of GPL.

Signnaling Pathway	Interventions	Experimental Model	Mechanism	PMID
MAPK	Curcumin	cisplatin (DDP)-induced mice	Suppress JNK1/2, ASK1, P38, JUN Enhance ERK1/2 and C-Myc	36178099
	Rhein	*H. pylori*-induced mice	Regulate TNF-α,COX-2,IL-6,IL-1β and Nrf2	36789982
	Panax Notoginseng Saponins	MMNG-induced rats	Regulate TLR2, TLR4/MAPK/NF-κB/iNOS	/
JAK/STAT3	Calycosin	*H. pylori*-induced rats	Regulate the integrin β1/NF-κB/DARPP-32 Inhibit STAT3	32606591
	Danggui Shaoyao Powder	*H. pylori*-induced rats	Up-regulate SOCS3 Down-regulate TLR4, p-JAK2, p-STAT3, NF-κB, MyD88, NLRP3, Bax, Bad	36046903
PI3K/AKT/mTOR	Berberine	MMNG-induced rats	Down-regulate TGF-β1, PI3K, p-Akt/Akt, p-mTOR/mTOR P70S6K Promote PTEN, LC3-II Beclin-1	33841162
	Epigallocatechin	MNNG and sodium salicylate-induced rats	Upregulate caspase-3, PTEN Reduce PI3K, Akt, mTOR	33628319
	Rg3	Atp4a-induced mice	Regulate PI3K, AKT, mTOR, HIF-1*β*, LDHA, HK-II	32076440
	Xiaojianzhong decoction	MNNG compound(MNNG, hot ranitidine-salt solution and 20% ethanol) induced rats	Decrease PI3K/AKT/mTOR Inhibit the p53/AMPK, ULK1 Ser-317, Ser-555	37009319
Hippo	Radix curcumae extract	MMNG-induced rats	Down-regulate VEGF, COX-2, PCNA	20210736
	Red Ginseng extract	*H. pylori*-induced Mongolian gerbils	Suppress KC, IL-1β iNOS	24558304
	Huazhuo Jiedu formula	MMNG-induced rats	Down-regulate TAZ Up-regulate LATS2 and MST1	/
Hedgehog	Modified Gualou Xiebai Banxia Decoction,	MMNG-induced rats	Suppress JAK2/STAT3 Promote Hedgehog	/
	Weiweikang	Sodium-salicylate-induced rats	Suppress Smo、Shh and SuFu	/
	Spleen-fortifying, fire-clearing and collateral-unblocking medicinals	MNNG compound(MNNG, hot ranitidine-salt solution and 20% ethanol) induced rats	Improve IL-1β, GAS Reactivate Hedgehog signal pathway	/
Wnt/β-catenin	Dendrobium officinale polysaccharide	MMNG-induced rats	Downregulate Wnt2β, Gsk3β, PCNA, CyclinD1, β-catenin	31340453
	Liquiritigenin	MMNG-induced rats	Decrease Wnt1, β-catenin, cyclin D1 Increased GSK-3β	34194556

### 5.1 MAPK signaling pathway

The activation of MAPK signaling pathways involves three main components: MAP3K, MAP2K, and MAPK. MAPK contains P38, JNK, ERK. Once MAPKs are activated, they go on to stimulate various substrate proteins, thereby regulating a wide range of cellular activities (88). The p38 signal regulates the activation of ROS and EMT. ROS, in turn, can activate EGFR, thereby initiating the Ras/MAPK pathway and participating in activating NF-κB and COX-2. Ultimately, this intricate signaling cascade promotes the progression of GPL cell canceration (89). Curcumin, a phenolic compound renowned for its robust antioxidant properties, effectively mitigates cisplatin-induced inflammation and apoptosis in the gastric mucosa by modulating the NF-κB and MAPKs signaling pathways (90). Rhein exerts anti-inflammatory and antioxidant effects in CAG. It can improve the mouse model of CAG infected with *H.pylori* by inhibiting inflammation and oxidative stress. The repair of gastric mucosal damage is achieved through the activation of Nrf2 and MAPK signaling (91). Panax Notoginseng Saponins have the ability to stimulate the JNK signaling pathway, trigger apoptosis, suppress inflammatory responses, slow down the malignant progression of gastric mucosa, and provide protective benefits for the gastric mucosa (92).

### 5.2 JAK/STATs signaling pathway

In the JAK/STATs pathway, *H.pylori* elevates levels of inflammatory factors such as IL-6, stimulate the excessive activation of JAK/STATs signaling, leading to the progression of GPL. Once activated, JAKs proceed to phosphorylate the primary substrate Signal Transducers and Activator of Transcription (STATs). Subsequently, STATs molecules form dimers, which are subsequently transported to the nucleus. Within the nucleus, these dimers bind to specific regulatory sequences, thereby regulating the transcription of target genes. One such example of target genes is the Suppressor of Cytokine Signaling (SOCS) family, which can be activated or suppressed by this intricate signaling pathway, leading to the progression of GPL (93, 94). Normally, interferon gamma activates JAK/STATs to participate in the immune response. In the early stages of *H. pylori* infection, cholesterol in the gastric epithelial cells can be consumed, preventing interferon gamma signaling from activating the JAK/STAT signaling pathway and thus evading the immune response (95). However, in the progression of GPL to gastric cancer, STATs increased with the cascade of GPL to gastric cancer, which possible mechanism is the activation of PD-L1, which led to the progression of gastric cancer (93). Calycosin is a flavonoid derived from the root of Astragalus membranaceus, known for its antioxidant and anti-inflammatory properties. It prevents gastric mucosal damage in MNNG-induced GPL rats by inhibiting STAT3 expression in GPL (96). Danggui Shaoyao Powder has been found to up-regulate SOCS3 protein levels, down-regulate TLR4, p-JAK2, p-STAT3 and NF-κB protein levels, and reduce gastric mucosal atrophy in rats (97).

### 5.3 PI3K/AKT/mTOR signaling pathway

The PI3K/AKT/mTOR signaling pathway is closely linked to apoptosis and autophagy (98). *H.pylori* and inflammatory factors trigger RAS, subsequently stimulating downstream PI3K, in turn, facilitates the activation of AKT through phosphorylation and is drawn to the cell membrane by AKT. This activates mTOR complex 1 (mTORC1). The effectors of mTORC1, including proteins like HIF1a and PGC-1a, play a pivotal role in regulating various cellular functions associated with oncogenic phenotypes (99). Several natural antioxidants have been reported to be effective for GPL via regulation of this signaling pathway. For example, berberine has been shown to down-regulate TGF-β1, PI3K/AKT/mTOR signaling, and P70S6K, while promoting PTEN, LC3-II, and Beclin-1, ultimately leading to an improvement in CAG (100). Epigallocatechin Gallate (EGCG), a natural antioxidant abundant in tea, has been found to improve GPL (101). Moreover, Ginsenoside Rg3, a natural compound found in *Ginseng*, has gained attention for anti-inflammatory, antioxidant, and anticancer properties. Ginsenoside Rg3 has been demonstrated to regulate PI3K/AKT/mTOR and HIF-1α. Ginsenoside Rg3 can be used to induce apoptosis and treat GPL (102). Xiaojianzhong decoction has been shown to reduce gastric mucosal hypoxia, regulate the PI3K/AKT/mTOR pathway to improve GPL (103).

### 5.4 The Hippo pathway

In the signal cascade of the Hippo pathway, there exist two states known as “Hippo on” and “Hippo off.” In the “Hippo on” state, the transcription factors, YAP/TAZ can associate with the 14-3-3 protein complex and become sequestered in the cytoplasm, ultimately undergoing degradation via ubiquitination. However, in the “Hippo off” state, YAP/TAZ cannot bind to the 14-3-3 complex, enabling their entry into the nucleus where they participate in complex formation, regulate the downstream target (104). The Hippo is link to inflammatory endothelial injury. Study has found Red Ginseng extract has also been found to inhibit IL-1β and iNOS associated with *H.pylori* infection, suppress the phosphorylation of IκBα, and reduce the increase of gastric mucosal LPO level and MPO activity, thereby delaying the evolution of GPL (105). Additionally, radix curcumae extract has been shown inhibit VEGF, COX-2, offering a potential treatment for GPL (106). Huazhuo Jiedu formula has been shown to down-regulate the expression of Hippo/TAZ signaling pathway and its related protein transcriptional coactivators PDZ-binding motif (TAZ), tumor suppressor kinase (LATS2), and mammalian sterile line 20-like kinase (MST1) levels in gastric mucosa tissue, thereby improving chronic atrophic gastritis (107).

### 5.5 Hedgehog signaling pathway

Hedgehog signaling starts with by various Hedgehog ligands, such as Shh, Ihh, and Dhh, which bind to membrane-bound receptor known as Patched. Interestingly, Patched receptors have shown to promote the progression of GC (108). Inflammation can accelerate the expression of the Hedgehog signaling pathway and induce IFNα, which regulates the level of SLFN4 and leads to atrophic gastritis in infected gastric mucosa (109). Modified Gualou Xiebai Banxia Decoction, a traditional Chinese medicine prescription, promotes the Hedgehog pathway to improve the inflammatory activity in rats with CAG (110). The Chinese patent medicine Weiweikang can regulate the levels of Smo, Shh, and SuFu proteins in the gastric mucosa of rats, which improves the inflammatory changes in the gastric mucosa and treats GPL (111). Spleen-fortifying, fire-clearing and collateral-unblocking medicinals affects the up-regulation of Shh, Ptch1, Smo, and Gli1 protein expression, the down-regulation of Gli2, Gli3, and Sufu protein expression, reduces serum IL-1β levels, and improves the pathological changes in CAG rats (112).

### 5.6 Wnt/β-catenin pathway

In the Wnt/β-catenin pathway, *H.pylori* infection activates the levels of Wnt pathway-related proteins as gastric disease progresses (113). Under the action of Wnt ligands, CK1α, Axin, GSKβ and LRP5/6 are recruited to form a complex. This results in an increase in large amounts of free beta-catenin, which increases the progression of gastric disease (114). Traditional Chinese medicine can intervene GPL by regulating the Wnt/β-catenin pathway. Dendrobium officinale polysaccharide can reduce Wnt2β and β-catenin to inhibit the progression of GPL (115). Liquiritigenin and hesperidin in Jianpiyiqi formula can improve mucosal atrophy and inflammation and help treat GC by down-regulating Wnt1, β-catenin and up-regulating GSK-3β (116).

## 6 Challenges and future perspectives

The inflammatory microenvironment in GPL has shed light on the crucial part of inflammation in the progression of GC. However, several challenges need to be addressed to achieve a comprehensive understanding of this complex process and develop effective prevention and treatment strategies. Here we discuss some of the key challenges and propose future perspectives in this field.

One major challenge in studying the inflammatory microenvironment in GPL is the heterogeneity of these lesions. Though GPL provides a critical stage for clinical intervention of GC, GPL can vary in terms of histopathological features, molecular alterations, and inflammatory cell infiltration patterns. It is central to uncover the underlying mechanisms driving this heterogeneity and identifying specific biomarkers for GPL. Nowadays, application of single-cell sequencing and imaging methods to explore the cellular heterogeneity within GPL, will shed light on unraveling distinct subpopulations of inflammatory cells, and understanding their functional diversity and interaction patterns (117).

Another big challenge lies in deciphering the intricate interplay between immune cells and inflammation in GPL progression. Immune cells and cytokines play an important role in the transformation of GPL. Their interactions and communication, along with their specific contributions to GPL development, remain unclear. It is also necessary to delineate the dynamic interactions between immune cells and the inflammatory microenvironment, which may provide novel targets for immunotherapeutic approaches (118).

The inflammatory microenvironments are common pathological features that drive the development of multiple chronic diseases such as cancer. In GPL, abnormal activation of inflammatory microenvironment has been shown to be link to the progression of the disease. However, it is important to recognize that the inflammatory microenvironment consists of a complex network of cells, molecules, and signaling pathways. Targeting inflammatory microenvironment by chemical interventions may disrupt the delicate balance necessary for normal physiological functions, leading to potential side effects and toxicity. Thus, the proper target should be rigorously validation *in vivo* before implementing any intervention strategies (119).

Traditional Chinese medicine and its active components have demonstrated distinct advantages in influencing the release of inflammatory factors and treating GPL (120). However, the underlying mechanisms how they affect the inflammatory microenvironment to prevent GPL, along with their true effect in GPL clinical trials, remain unclear. In the future, investigating the molecular mechanisms how TCM interventions modulate inflammatory microenvironment and exploring their synergistic effects via drug combinations, will pave the way for the integration of TCM into clinical practice.

Collectively, the challenges presented necessitate further research efforts. By addressing these challenges and exploring the proposed future perspectives, we can advance our understanding of the inflammatory microenvironment in GPL and develop effective strategies for the prevention and treatment of GPL.

## Author contributions

SZ: Writing – original draft. YS: Writing – original draft. HL: Writing – original draft. DZ: Writing – original draft. JF: Writing – review & editing. HP: Writing – review & editing. WL: Funding acquisition, Writing – review & editing.
